# Three Alveolar Phenotypes Govern Lung Function in Murine Ventilator-Induced Lung Injury

**DOI:** 10.3389/fphys.2020.00660

**Published:** 2020-06-30

**Authors:** Bradford J. Smith, Gregory S. Roy, Alyx Cleveland, Courtney Mattson, Kayo Okamura, Chantel M. Charlebois, Katharine L. Hamlington, Michael V. Novotny, Lars Knudsen, Matthias Ochs, R. Duncan Hite, Jason H. T. Bates

**Affiliations:** ^1^Department of Bioengineering, College of Engineering, Design & Computing, University of Colorado Denver | Anschutz Medical Campus, Aurora, CO, United States; ^2^Department of Pediatric Pulmonary and Sleep Medicine, School of Medicine, University of Colorado Anschutz Medical Campus, Aurora, CO, United States; ^3^Vermont Lung Center, Larner College of Medicine, The University of Vermont, Burlington, VT, United States; ^4^Lerner Research Institute, Cleveland Clinic, Cleveland, OH, United States; ^5^Institute of Functional and Applied Anatomy, Hannover Medical School, Hanover, Germany; ^6^Biomedical Research in Endstage and Obstructive Lung Disease Hannover (BREATH), Member of the German Center for Lung Research, Hanover, Germany; ^7^Institute of Functional Anatomy, Charité Medical University of Berlin, Berlin, Germany; ^8^Division of Pulmonary, Critical Care and Sleep Medicine, Department of Internal Medicine, College of Medicine, University of Cincinnati, Cincinnati, OH, United States

**Keywords:** ventilator-induced lung injury, stereology, pulmonary surfactant, lung function, alveolar mechanics

## Abstract

Mechanical ventilation is an essential lifesaving therapy in acute respiratory distress syndrome (ARDS) that may cause ventilator-induced lung injury (VILI) through a positive feedback between altered alveolar mechanics, edema, surfactant inactivation, and injury. Although the biophysical forces that cause VILI are well documented, a knowledge gap remains in the quantitative link between altered parenchymal structure (namely alveolar derecruitment and flooding), pulmonary function, and VILI. This information is essential to developing diagnostic criteria and ventilation strategies to reduce VILI and improve ARDS survival. To address this unmet need, we mechanically ventilated mice to cause VILI. Lung structure was measured at three air inflation pressures using design-based stereology, and the mechanical function of the pulmonary system was measured with the forced oscillation technique. Assessment of the pulmonary surfactant included total surfactant, distribution of phospholipid aggregates, and surface tension lowering activity. VILI-induced changes in the surfactant included reduced surface tension lowering activity in the typically functional fraction of large phospholipid aggregates and a significant increase in the pool of surface-inactive small phospholipid aggregates. The dominant alterations in lung structure at low airway pressures were alveolar collapse and flooding. At higher airway pressures, alveolar collapse was mitigated and the flooded alveoli remained filled with proteinaceous edema. The loss of ventilated alveoli resulted in decreased alveolar gas volume and gas-exchange surface area. These data characterize three alveolar phenotypes in murine VILI: flooded and non-recruitable alveoli, unstable alveoli that derecruit at airway pressures below 5 cmH_2_O, and alveoli with relatively normal structure and function. The fraction of alveoli with each phenotype is reflected in the proportional changes in pulmonary system elastance at positive end expiratory pressures of 0, 3, and 6 cmH_2_O.

## Introduction

Mechanical ventilation is required for major surgery and resuscitation and in critical emergencies such as acute respiratory distress syndrome (ARDS) (Force et al., 2012), but this lifesaving therapy carries the risk of ventilator-induced lung injury (VILI). The mechanisms of VILI are well documented and include direct tissue injury from overdistension (volutrauma) (Webb and Tierney, 1974; Hernandez et al., 1989) and the cyclic collapse and reopening of small airways and alveoli (atelectrauma) (Muscedere et al., 1994). These injurious forces act at the microscale to damage the epithelial and endothelial cells that comprise the blood-gas barrier (Dreyfuss and Saumon, 1998; Hamlington et al., 2018b), allowing the ingress of proteinaceous edema into the distal airspace that is associated with changes in lung mechanics (Hamlington et al., 2016a).

Changes in the lung structure–function relationship during VILI are currently understood through the lens of organ-scale imaging techniques such as CT (Yen et al., 2019). However, the positive feedback mechanism between leak, surfactant function, altered mechanics, and injury occurs at the alveolar and acinar scales (Knudsen et al., 2018). As such, measurements of morphometry are necessary to bridge studies of cellular injury and alveolocapillary barrier disruption (Hamlington et al., 2018a) to organ-scale measurements of structure and function. Understanding how these mechanisms are associated across length scales is critical for developing approaches to mitigating them. Structure-function correlations may also improve clinical practice by defining the mechanistic basis for diagnostic parameters such as the driving pressure (Aoyama et al., 2018; Pereira Romano et al., 2020) and subject-specific computational models (Hamlington et al., 2016b; Mellenthin et al., 2019; Morton et al., 2019) that seek to optimize ventilation based on patient-specific alveolar mechanics.

To address this knowledge gap, we used design-based stereology to measure parenchymal structure over a range of air inflation pressures in a mouse model of VILI. The state of the pulmonary surfactant system was assessed by measuring minimum surface tension and surfactant composition. Organ-scale mechanical function was determined with the forced oscillation technique (FOT) to quantify stiffness, airway resistance, and tissue damping at different levels of lung inflation. Together, these data define the structure–function relationship in murine VILI.

## Materials and Methods

### Animal Procedures

Eight to ten week old female BALB/c mice (Jackson Laboratories, Bar Harbor, ME, United States) weighing 17.1–22.3 g were studied under Institutional Animal Care and Use Committee (IACUC)-approved protocols. Mice were anesthetized with an intraperitoneal (IP) injection of 100 mg/kg ketamine and 16 mg/kg xylazine, tracheostomized with a 18 ga metal cannula, and ventilated using a flexiVent small animal ventilator (SCIREQ, Montreal, QC, Canada). Respiratory drive was suppressed *via* 0.8 mg/kg pancuronium bromide administered at the onset of mechanical ventilation. Alternating doses of 50 mg/kg ketamine and 50 mg/kg ketamine with 8 mg/kg xylazine were administered at 30-min intervals with 150 μL IP 5% dextrose lactated Ringer’s solution. Fluid volume from the anesthetic was 10 μL/g. The electrocardiogram was monitored to assure that the rodents maintained a deep plane of anesthesia during paralysis.

Mechanical ventilation experiments to generate tissue for structural analysis were conducted at the University of Vermont and approved by the University of Vermont Institutional Animal Care and Use Committee (IACUC #14-056). Bronchoalveolar lavage fluid for surfactant analysis was collected under identical ventilation conditions by the same investigator at the University of Colorado Denver in experiments approved by the University of Colorado Denver Anschutz Medical Campus, IACUC (#00230).

### Ventilation Protocol

All animals were ventilated for a 10-min stabilization period with a delivered tidal volume (Vt) = 10 ml/kg at a respiratory rate (RR) = 200 breaths/min and a positive end expiratory pressure (PEEP) = 3 cmH_2_O (baseline ventilation). Recruitment maneuvers (RM) consisting of a 3 sec ramp to 30 cmH_2_O followed by a 3 sec breath hold were applied to reopen regions that may have collapsed during the surgical procedure. Following the stabilization period, lung function was assessed. Testing consisted of an RM and a dynamic pressure-volume (PV) loop that ramped airway pressure (Paw) from 0 to 37.5 cmH_2_O, held pressure for 3 sec, and ramped pressure back to zero over 3 sec. Derecruitability tests were then performed at PEEP = 6, 3, and 0 cmH_2_O, denoted by $D_{rec}^{PEEP6}$, $D_{rec}^{PEEP3}$, and $D_{rec}^{PEEP0}$. Each test consisted of an RM and then Vt = 10 ml/kg and RR = 200 breaths/min at the specified PEEP. Nine multi-frequency FOT impedance measurements were recorded at 18 s intervals and fit to the constant phase model (Hantos et al., 1992) to determine respiratory system elastance (H), tissue damping (G), and Newtonian resistance (Rn). The total duration of the lung function assessment was 13 min. Lungs from the control group were then harvested for morphometric analysis. In a separate sub-group of animals, bronchoalveolar lavage fluid (BALF) was collected for surfactant analysis.

The VILI group was then subjected to repeated blocks of ventilation consisting of 4 min 45 s of injurious ventilation with a plateau pressure (Pplat) = 37.5 cmH_2_O, RR = 50 breaths/min, inspiratory:expiratory ratio of 1:2, and PEEP = 0 cmH_2_O. This Pplat was selected to generate severe VILI in an experimentally tractable timeframe without causing any pneumothoraces. At the end of each block a PV loop was recorded followed by two FOT measurements separated by 10 s of baseline ventilation. These 5-min ventilation blocks were applied until *H* doubled, at which point the lung function assessment was repeated prior to harvest for either morphometry or surfactant analysis. The mean duration of injurious mechanical ventilation was 87.3 min with a standard deviation of 17.5 min. Including with the 10 min stabilization period and two 13 min lung function assessments, the mean duration of ventilation for the VILI group was 123.3 min.

### Surfactant Analysis

Bronchoalveolar lavage fluid was collected by instilling and suctioning back 1 ml warm normal saline three times and then centrifuging at 700 *g* at 4°C for 5 min. A total of 100 μl of the cell-free supernatant for each animal was separated, and the remainder of the BALF from three mice in the same treatment group was combined and centrifuged at 40,000 *g*. The ultracentrifuged supernatant containing the surfactant small aggregate (SA) was stored at −80°C for later analysis. The surfactant pellet, containing the large aggregate (LA) fraction, was washed twice by resuspending the pellet in 1 ml saline and centrifuging at 40,000 *g*. Finally, the LA pellet was resuspended in 100 μl saline and stored at −80°C for later analysis.

Quantitation of the surfactant recovered from the BAL was completed by measuring lipid phosphorous content in the LA and SA fractions as previously described (Rouser et al., 1970). Total surfactant recovery represents the combined amount of both fractions. Total BAL protein was quantified with a BCA assay (ThermoShandon-Pierce). Surfactant surface lowering activity was assessed by measuring minimal surface tension of the LA fraction *ex vivo* using a pulsating bubble surfactometer (General Transco) as described (Enhorning and Holm, 1993; Hite et al., 2005). Surfactant was preheated to 37°C at 2 mg (PL from LA)/ml in 150 mM NaCl, 5 mM CaCl_2_, 5 mM Tris solution, and the samples were pulsated at 20 pulses/min for 20 min.

### Lung Tissue Preparation

Lung structure was analyzed in a separate subgroup of mice that were not subjected to a bronchoalveolar lavage. The lungs were fixed through the vasculature while the air inflation pressure was held at a prescribed level (described below) to maintain surface tension effects and allow comparison to the lung function data (Gil et al., 1979; Bachofen et al., 1982). The fixation process was initiated during baseline ventilation by performing a bilateral thoracotomy and flushing the pulmonary circulation with 5 ml 100 U/ml heparinized saline with 3% 100 kDa dextran at a pressure of 25 cmH_2_O. Three RMs were applied and then Paw increased to 30 cmH_2_O over 3 s, held for 3 s, decreased to either 2, 5, or 10 cmH_2_O, and the trachea was ligated. These pressures were selected based on our previous morphometric analysis of bleomycin-injured rats (Knudsen et al., 2018) where nearly all recruitable alveoli were open at Paw = 10 cmH_2_O and approximately half the recruitable alveoli were closed at Paw = 5 cmH_2_O. The lower limit of Paw = 2 cmH_2_O was selected since PEEPs < 2 cmH_2_O are not typically applied in lung-protective ventilation. Five animals were prepared at each airway pressure for the control and VILI groups. The lungs were then perfused with 1.5% glutaraldehyde, 1.5% paraformaldehyde in 0.15 M HEPES buffer for approximately 5 min before immersion fixation for at least 24 h.

Tissue processing and sampling was conducted using standardized protocols (Mühlfeld et al., 2013; Knudsen et al., 2018). Briefly, non-pulmonary tissue was removed, and the total lung volume determined using Archimedes principle. The first step of systematic uniform random sampling was conducted by cutting the lungs into 1.5 mm transverse sections and retaining either even or odd numbered slabs. The slabs were post-fixed with 1% OsO_4_ in 0.1 M cacodylate buffer for 2 h and then overnight in half saturated aqueous uranyl acetate, dehydrated in a graded acetone series, and embedded in glycol methacrylate (Technovit 7100, Heraeus Kulzer, Wehrheim, Germany) to avoid tissue shrinkage (Schneider and Ochs, 2014). The first and fourth 1.5 μm sections from a consecutive series were mounted and stained with orcein and then used with the physical disector stereology probe to estimate alveolar number. Another 1.5 μm section was stained with toluidine blue to estimate volumes and surface areas.

### Design-Based Stereology

The stereological analysis was conducted according to standards for lung structure quantification (Hsia et al., 2010). A majority of the systematic uniform random sampling and image acquisition was conducted using a Zeiss Axioscope II with a Ziess Axiocam AR camera (Zeiss, Thornwood, NY, United States) controlled with the Stereo Investigator software package (MBF Bioscience, Williston, VT, United States). Approximately 10% of the images (distributed randomly across groups and subjects) were recorded using an Olympus BX53 with a DP73 camera (Olympus, Waltham, MA, United States) controlled with the NewCast stereology software (Visiopharm, Hørsholm, Denmark). Stereological quantification of all images was performed using a custom MATLAB (Mathworks, Natick, MA, United States) graphical user interface.

A cascade sampling design was used starting with the volume fraction of parenchyma in the whole lung [Vv(par/lung)] that we define as areas contributing to gas exchange and excluding airways, vessels outside the septal walls, and peribronchiolar tissue. This, and all volume fractions, were measured using point counting. The percentage of the tissue section analyzed (the sampling fraction, *S*_F_) for Vv(par/lung) was 100% at 5× magnification. The parenchyma was then subdivided into volume fractions of alveolar airspace [Vv(alvair/par)], alveolar duct airspace [Vv(ductair/par)], and non-air material [Vv(non-air/par)] that include tissue and airspace edema fluid. This assessment was performed at 20× magnification and *S*_F_ = 11%. Fields of view were selected for analysis using systematic uniform random sampling whereby the morphometry software selected a random starting location and images were then automatically sampled on a 1,200 × 1,200 μm grid over the entire tissue section. This approach blinds the investigator to the selection of the images used for analysis, thus removing the potential for bias. The non-air material in the parenchyma [Vv(non-air/par)] was then analyzed at 40× magnification (*S*_F_ = 2.8%, 1,200 μm grid spacing) to determine the volume fractions of septal tissue of patent alveoli [Vv(sep,air/par], total septal tissue [Vv(sep,total/par)], and airspace edema [Vv(edema/par)]. The volume of each compartment (demarcated with V in place of Vv) was determined by multiplying the volume fractions by the volume of the reference space. The surface area per volume available for gas exchange [Sv(alvair/par)] was estimated at 40× magnification (*S*_F_ = 2.8%, 1,200 μm grid spacing) by counting line intersections with aerated septal tissue that was not covered with alveolar edema. The gas exchange surface area [S(alvair)] was determined by multiplying Sv(alvair/par) by the parenchymal volume [V(par)] and the mean septal thickness defined as τ(sep) = 2 V(sep,air)/S(alvair). The physical disector method (Ochs et al., 2004) was used to determine the number of patent [N(alv)] and flooded [N(flood)] alveoli that we define as alveoli with visible architecture and alveolar edema fully blocking the alveolar mouth using 40× magnification (*S*_F_ = 6.25%, 800 μm grid spacing). The total number of alveoli is then N(alv,total) = N(alv) + N(flood), the number-weighted average alveolar volume *V*_n_(alv) = V(alvair)/N(alv), and the alveolar surface area to volume ratio S/V(alv) = S(alvair)/V(alvair).

### Statistical Analysis

Data were curated in MATLAB and statistical analysis was performed in R (The R Foundation). A Shapiro–Wilk test was used to assess normality. If the data were not significantly different from the normal distribution (*p* > 0.05) then an ANOVA was performed followed by pairwise comparisons using the lsmeans package (Lenth, 2016) and Tukey’s adjustment for multiple comparisons. Data that failed the normality test was analyzed with a Kruskal–Wallis test followed by a rank-sum Conover–Iman test (PMCMR package) with the Holm correction for multiple comparisons.

The time series of lung function parameters H, G, and Rn measured during the derecruitability tests were analyzed using a linear mixed effects model (LME) (NLME package in R) on the natural logarithm of the time and parameter values. This is equivalent to fitting the equation *y* = α *t*^β^ to the time series of measurements as we have previously described (Smith et al., 2017). Fixed effects were derecruitability test PEEP $\left(D_{rec}^{PEEP}\right)$, the time within the derecruitability test (*t*), and whether the measurement was recorded before or after VILI (*Injury*). The intercept for each animal was included as a random effect and the Akaike information criterion (AIC) was used to determine the most appropriate model. Temporal autocorrelation was accounted for with a first-order autocorrelation structure for the continuous time covariate grouped by animal, derecruitability test PEEP, and injury status (Pinheiro and Bates, 2000).

## Results

**FIGURE 1:**
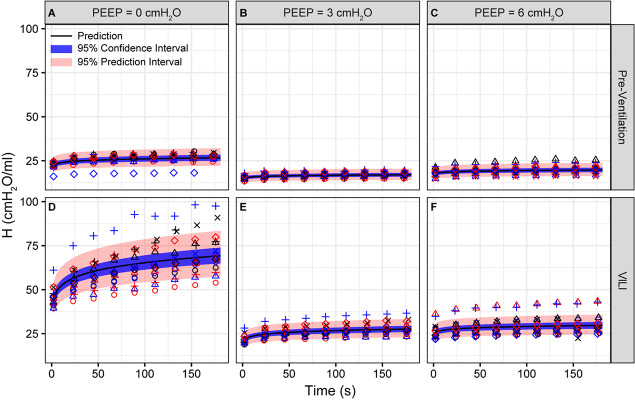
Pulmonary system elastance (H) measured during derecruitability tests at PEEP = 0 **(A,D)**, 3 **(B,E)**, and 6 cmH_2_O **(C,F)** before **(A–C)** and after injurious mechanical ventilation **(D–F)**. The LME model fit is shown with a black line, the 95% confidence interval is shown with a blue band, and the 95% prediction interval is shown with a red band. Symbols depict raw data for each animal and are consistent between Figures 1–3. Fifteen mice are in each group.

**FIGURE 2:**
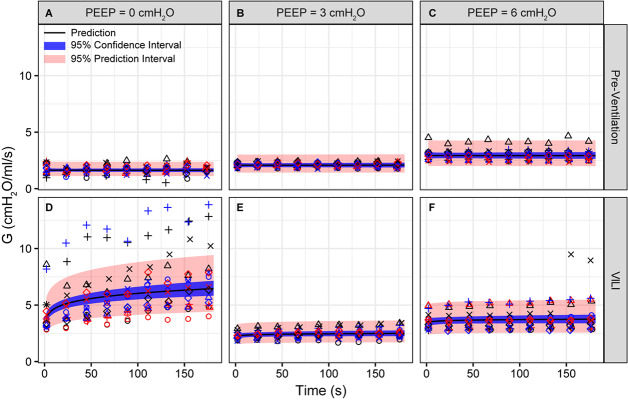
Tissue damping (G) measured during derecruitability tests at PEEP = 0 **(A,D)**, 3 **(B,E)**, and 6 cmH_2_O **(C,F)** (columns) before **(A–C)** and after injurious mechanical ventilation **(D–F)**. The LME model fit is shown with a black line, the 95% confidence interval is shown with a blue band, and the 95% prediction interval is shown with a red band. Symbols depict raw data for each animal and are consistent between Figures 1–3. Fifteen mice are in each group.

**FIGURE 3:**
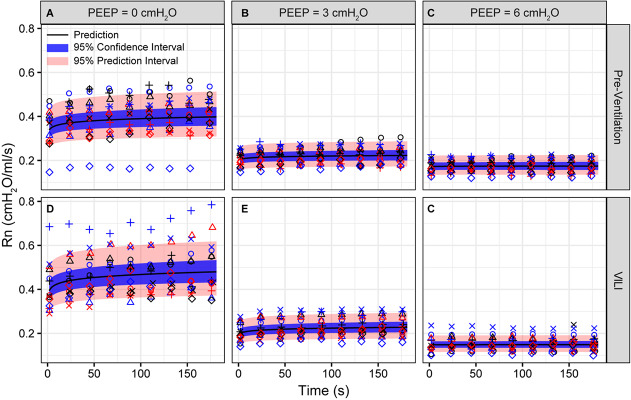
Newtonian resistance (Rn) measured during derecruitability tests at PEEP = 0 **(A,D)**, 3 **(B,E)**, and 6 cmH_2_O **(C,F)** before **(A–C)** and after injurious mechanical ventilation **(D–F)**. The LME model fit is shown with a black line, the 95% confidence interval is shown with a blue band, and the 95% prediction interval is shown with a red band. Symbols depict raw data for each animal and are consistent between Figures 1–3. Fifteen mice are in each group.

Pulmonary system elastance following an RM in the uninjured lungs (Figure 1, first row) was significantly different between the derecruitability tests at different PEEP levels, while the rate of change of H over time (the slope) remained consistent between PEEPs (Table 1). VILI (Figure 1, second row) increased the H intercept at all PEEPs with a proportionally greater increase occurring in $D_{rec}^{PEEP0}$ where the elastance at the start of the derecruitability test was approximately doubled. The rate of elastance increase over time was also elevated in $D_{rec}^{PEEP0}$ and, to a lesser extent, in $D_{rec}^{PEEP3}$. Tissue damping (G) increased monotonically with PEEP prior to injury (Figure 2, first row) and was independent of *t* (Table 2). Following VILI, G demonstrated a marked increase in $D_{rec}^{PEEP0}$ that was similar in magnitude to the change in H. Central airway resistance (Rn) tended to decrease with PEEP (Figure 3) in both healthy and injured lung. Following VILI, Rn was slightly increased in $D_{rec}^{PEEP0}$ and decreased in $D_{rec}^{PEEP6}$. The temporal dependence of Rn was not affected by VILI (Table 2).

**TABLE 1:**
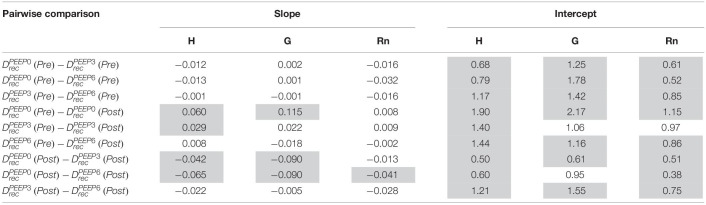
Pairwise comparison of the LME model using log-transformed lung function parameters, which is equivalent to fitting the equation *y* = α *t*^β^ to the raw data.

*The numeric values for slope indicate the change in β from the left-hand to the right-hand derecruitability test listed under **“**Pairwise Comparison.**”** The numeric values provided for the intercepts indicate the fold change in the raw (not log transformed) intercept α. Gray cells indicate *p* < 0.05. Fifteen mice are in each group.*

**TABLE 2:** Results from regression analysis predicting log-transformed H, G, or Rn from derecruitability test PEEP ($D_{rec}^{PEEP}$) with factors $D_{rec}^{PEEP0}$, $D_{rec}^{PEEP3}$, and $D_{rec}^{PEEP6}$; the log-transformed continuous time within the derecruitability test (*t*); and an *Injury* factor describing if the measurements were recorded at the start of the experiment (pre-ventilation) or after ventilation to a doubling of pulmonary system elastance (VILI).

	H		G		Rn	
Predictor	Estimate (SE)	*p*-value	Estimate (SE)	*p*-value	Estimate (SE)	*p*-value
Intercept	3.083 (0.034)	<0.0001	0.500 (0.062)	<0.0001	−1.103 (0.057)	<0.0001
*t*	0.038 (0.006)	<0.0001	−0.002 (0.015)	0.8857	0.035 (0.009)	0.0002
$D_{rec}^{PEEP3}$	−0.386 (0.036)	<0.0001	0.228 (0.075)	0.0024	−0.499 (0.009)	<0.0001
$D_{rec}^{PEEP6}$	−0.229 (0.036)	<0.0001	0.580 (0.075)	<0.0001	−0.657 (0.047)	<0.0001
VILI	0.642 (0.036)	<0.0001	0.778 (0.075)	<0.0001	0.146 (0.047)	0.0021
$t\times D_{rec}^{PEEP3}$	−0.012 (0.008)	0.1529	0.002 (0.021)	0.8910	−0.016 (0.013)	0.2292
$t\times D_{rec}^{PEEP6}$	−0.014 (0.008)	0.1088	0.001 (0.021)	0.9627	−0.032 (0.013)	0.0138
*t*×VILI	0.060 (0.008)	<0.0001	0.115 (0.021)	<0.0001	0.008 (0.013)	0.5550
$D_{rec}^{PEEP3}\times \text{VILI}$	−0.309 (0.051)	<0.0001	−0.710 (0.106)	<0.0001	−0.176 (0.067)	0.0089
$D_{rec}^{PEEP6}\times \text{VILI}$	−0.279(0.051)	<0.0001	−0.623 (0.106)	<0.0001	−0.298 (0.067)	<0.0001
$t\times D_{rec}^{PEEP3}\times \text{VILI}$	−0.031 (0.012)	0.0100	−0.093 (0.030)	0.0015	0.002 (0.019)	0.9034
$t\times D_{rec}^{PEEP6}\times \text{VILI}$	−0.051 (0.012)	<0.0001	−0.097 (0.030)	0.0009	−0.009 (0.019)	0.6143

*Fifteen mice are in each group.*

**FIGURE 4:**
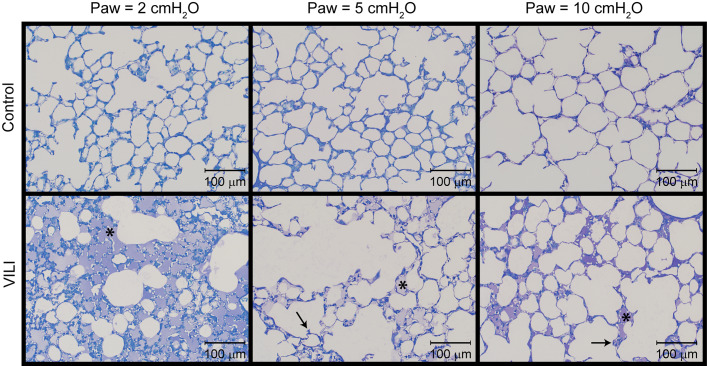
Representative micrographs for control (first row) and VILI (second row) mice that were perfusion fixed at an airway pressure Paw = 2 cmH_2_O (first column), Paw = 5 cmH_2_O (second column), and Paw = 10 cmH_2_O (third column). Asterisk indicates alveolar edema and arrows denote retracted alveolar septa.

The changes in lung function are associated with structural alterations in the perfusion-fixed lung tissue. Alveoli in the Control lungs (Figure 4, first row) remained patent from Paw = 10 cmH_2_O (right column) down to airway pressures of 2 cmH_2_O (left column). Note that these airway pressures are applied in open-chested mice, and the contributions of the chest wall are discussed below. By contrast, the VILI lungs exhibited diffuse alveolar edema (second row, asterisks) and retraction of the alveolar septa (second row, arrows) at Paw = 5 and 10 cmH_2_O. At Paw = 2 cmH_2_O, the injured parenchyma tended to consolidate into patches of flooded and collapsed alveoli with air remaining in the ductal space. These injured regions were interspersed between areas of parenchyma that appeared normal.

**FIGURE 5:**
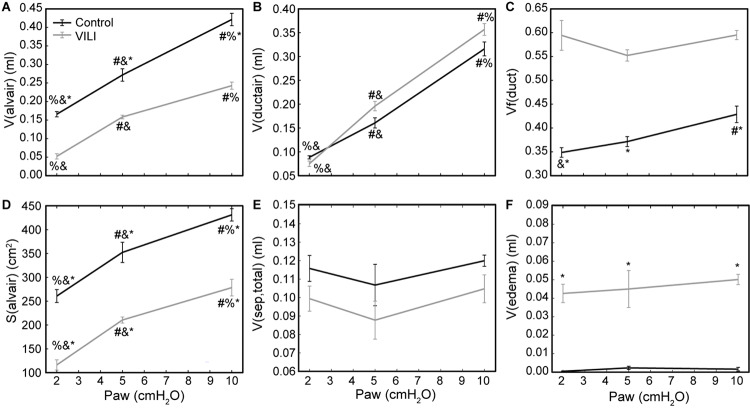
Volume of air in the alveoli V(alvair) **(A)**, volume of air in the alveolar ducts V(ductair) **(B)**, volume fraction of parenchymal air in the alveolar ducts Vf(duct) **(C)**, gas-exchanging surface area S(alvair) **(D)**, total septal tissue volume V(sep,tot) **(E)**, and alveolar edema volume V(edema) **(F)** for the control (black lines) and VILI (gray lines) mice fixed at airway pressures Paw = 2, 5, and 10 cmH_2_O. Significant differences from Paw = 2 cmH_2_O (#), Paw = 5 cmH_2_O (%), Paw = 10 cmH_2_O (&) in the same treatment group. Significant difference at the same airway pressure between treatment groups is shown with asterisks. *n* = 5 for each point and the error bars show standard error.

**FIGURE 6:**
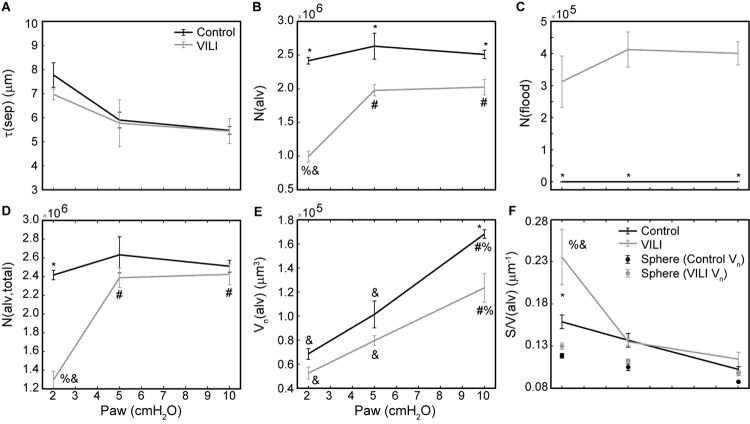
Average alveolar septal wall thickness **(A)**, number of patent alveoli N(alv) **(B)**, number of flooded alveoli N(flood) **(C)**, total number of alveoli with discernable structure N(alv,total) = N(alv) + N(flood) **(D)**, and the number-weighted mean alveolar volume V_n_(alv) **(E)** for the control (black lines) and VILI (gray lines) mice fixed at airway pressures Paw = 2, 5, and 10 cmH_2_O. Points plotted with the alveolar surface area to volume ratio [S/V(alv), **(F)**] depict the S/V for a sphere with volume *V*_n_(alv). Significant differences from Paw = 2 cmH_2_O (#), Paw = 5 cmH_2_O (%), Paw = 10 cmH_2_O (&) in the same treatment group. Significant difference at the same airway pressure between treatment groups is shown with asterisks. *n* = 5 for each point and the error bars show standard error.

Quantitative structural changes with VILI included a significant decrease in V(alvair) (Figure 5A) in conjunction with reduced N(alv) (Figure 6B). The average alveolar volume [V_n_(alv)] is defined as the ratio of these two quantities and indicates a modest stiffening of patent alveoli with injury (Figure 6E). By contrast, V(ductair) did not demonstrate significant alterations in VILI (Figure 5B), leading to a fractional shift of ventilation volume from the alveoli to the alveolar ducts with VILI (Figure 5C) that was significant at all airway pressures. The surface area available for gas exchange (Figure 5D) was significantly reduced across all airway pressures in VILI. This reduction is primarily due to a loss of ventilated alveoli and not to reduced surface area to volume ratio in the remaining patent units as indicated by increased S/V(alv) when comparing control and VILI at Paw = 2 cmH_2_O (Figure 6F, lines). For comparison, Figure 6F shows the hypothetical surface area to volume ratios for perfectly spherical alveoli (symbols), and these values are less than those measured *in vivo* despite the reduction in area caused by the alveolar mouth. The increase in S/V(alv) at low Paw in VILI suggests that the alveolar geometry becomes more distorted in the injured lung. Injurious ventilation did not change V(sep, total) (Figure 5E) or τ(sep), and although septal thickness tended to increase with decreasing Paw there were no significant alterations (Figure 6A). Little to no airspace edema was observed in the control animals (Figure 5F), and V(edema) was constant across inflation pressures in VILI.

The number of ventilated alveoli [N(alv), Figure 6B] remained constant across inflation pressures in the Control animals, indicating an absence of derecruitment even at Paw = 2 cmH_2_O. The VILI mice demonstrated a constant N(alv) at Paw = 5 and 10 cmH_2_O that was significantly less than in the Controls. Reducing the inflation pressure to 2 cmH_2_O in the VILI animals led to a significant reduction in aerated alveoli. Flooded alveoli [N(flood), Figure 6C] were defined as having a liquid meniscus fully spanning the alveolar mouth. N(flood) remained constant across inflation pressures in the VILI group, and no flooded alveoli were observed across the 15 control lungs analyzed (Table 3). Figure 6D shows that the total number of flooded and aerated alveoli [N(alv,total)] is consistent across all groups except at Paw = 2 cmH_2_O where there is a striking decrease with VILI.

**TABLE 3:** Summarized stereological data from control and VILI lungs.

Paw cmH_2_O	V(alvair) ml	V(ductair) ml	V(edema) ml	V(sep,air) ml	V(sep,total) ml	S(alvair) cm^2^	τ (sep) μm	N(alv)	N(flood)	N(alv,total)	V_n_(alv) μm^3^
**Control**											
2	0.166^%,&,^* (0.016)	0.089^%,&^ (0.007)	0.000* (0.000)	0.101* (0.019)	0.116 (0.016)	260.6^%,&,^* (30.4)	7.8 (1.1)	2,416,000* (110,815)	0* (0)	2,416,000* (110,815)	68,660^&^ (10,074)
5	0.272^#,&,^* (0.037)	0.161^#,&^ (0.024)	0.002* (0.002)	0.105* (0.025)	0.107 (0.025)	352.3^#,&,^* (47.4)	5.9 (0.7)	2,632,000* (431,010)	0* (0)	2,632,000 (431,010)	101,420^&^ (25,039)
10	0.421^#,%,^* (0.038)	0.316^#,%^ (0.032)	0.002* (0.002)	0.118* (0.008)	0.120 (0.007)	431.1^#,%,^* (29.1)	5.5 (0.3)	2,510,000* (140,712)	0* (0)	2,510,000 (140,712)	168,200^#,%,^* (8,106)
**VILI**											
2	0.052^%,&,^* (0.016)	0.075^%,&^ (0.013)	0.043* (0.011)	0.040* (0.009)	0.099 (0.015)	116.1^%,&,^* (24.0)	6.9 (0.5)	991,800^%,&,^* (182,486)	312,020* (178,976)	1,294,000^%,&,^* (202,745)	52,540^&^ (11,141)
5	0.158^#,&,^* (0.011)	0.196^#,&^ (0.022)	0.045* (0.022)	0.062* (0.027)	0.088 (0.023)	210.5^#,&,^* (14.1)	5.8 (2.2)	1,976,000^#,^* (184,201)	412,200* (122,873)	2,386,800^#^ (235,812)	79,540^&^ (9,362)
10	0.243^#,%,^* (0.022)	0.357^#,%^ (0.027)	0.050* (0.006)	0.075* (0.015)	0.105 (0.017)	278.4^#,%,^* (39.0)	5.4 (1.2)	2,024,000^#,^* (259,962)	400,400* (81,048)	2,423,400^#^ (247,958)	123,520^#,%,^* (26,696)

*Lungs were perfusion fixed at airway pressures Paw = 2, 5, and 10 cmH_2_O. Values are given as mean (standard deviation). Significant differences from Paw = 2 cmH_2_O (#), Paw = 5 cmH_2_O (%), Paw = 10 cmH_2_O (&) in the same treatment group. Significant difference at the same airway pressure between treatment groups is shown with asterisks. Each pressure in control and VILI includes n = 5 mice.*

**FIGURE 7:**
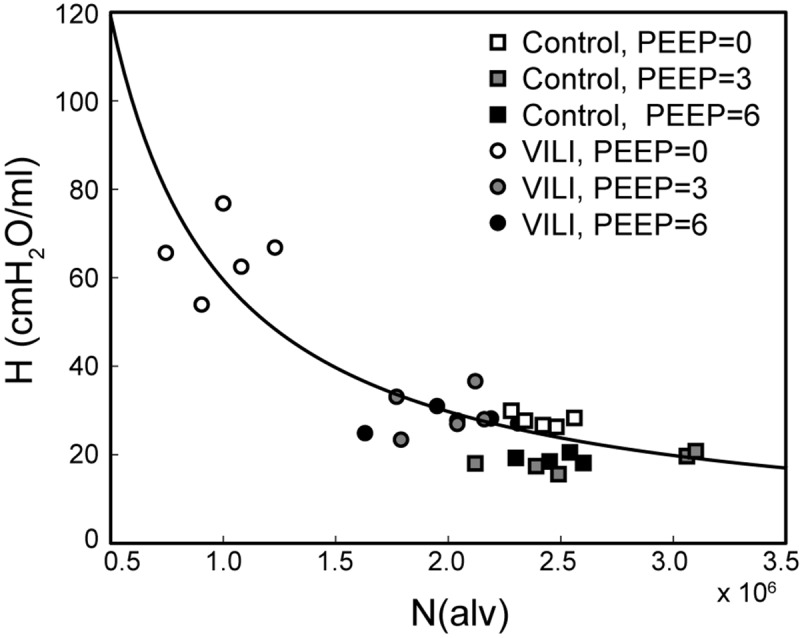
Pulmonary system elastance *H* at the end of $D_{rec}^{PEEP0}$ plotted against the number of alveoli N(alv) at Paw = 2 cmH_2_O (white symbols, *n* = 10) for control (squares) and VILI (circles). Gray symbols (*n* = 10) show the final elastance measurement in $D_{rec}^{PEEP3}$ against N(alv) at Paw = 5 cmH_2_O (*n* = 10); black symbols show the final elastance measurement in $D_{rec}^{PEEP6}$ against N(alv) at Paw = 10 cmH_2_O. The best-fit line (*R*^2^ = 0.78, *p* < 10^–13^) has the equation *H* = 24.40/(N(alv)/2.44 × 10^6^).

Figure 7 shows the pairwise relationships between the last elastance measurement in $D_{rec}^{PEEP0}$, $D_{rec}^{PEEP3}$, and $D_{rec}^{PEEP6}$ and N(alv) at Paw = 2, 5, and 10 cmH_2_O. Note that the mean pressure of the derecruitability tests is approximately 2–3 cmH_2_O greater than the onset pressures so that the mechanics and structure are measured at roughly the same pressure. Elastance is strongly correlated with the number of patent alveoli (*R*^2^ = 0.78), and the form of the regression was selected based on our prior computational simulations where elastance increases with the inverse of the lung open fraction (Smith et al., 2013, 2015; Hamlington et al., 2016b; Mellenthin et al., 2019).

**FIGURE 8:**
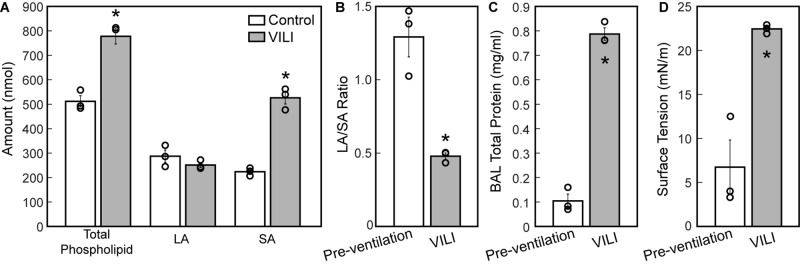
Total phospholipid content (**A**, white bars) is increased following ventilation and was accompanied by an increase in small aggregate (SA) and slight decrease in large aggregate (LA) causing a decrease in the LA/SA ratio **(B)**. The total protein content in the BAL **(C)** is significantly increased in the VILI animals and the minimum surface tension measured using the pulsating bubble surfactometer (PBS) was significantly higher post-injury. Significant differences are indicated with asterisks and individual datapoints representing the pooled analysis of three animals are indicated with circles. BALF from three mice was pooled for each of the three datapoints. Error bars show standard error.

The changes in lung structure and function are accompanied by alterations in the pulmonary surfactant system. BALF total phospholipid (PL) content (Figure 8A) significantly increased following high tidal volume ventilation due primarily to an increase in SA, which are less surface active. The increase in SA fraction also resulted in a striking and significant decrease in the LA/SA ratio (Figure 8B), a frequent marker of surfactant degradation, turnover, and/or injury. The fraction of the most surface-active LA was unchanged after VILI, but the functional quality of the LA pellet was significantly disrupted as demonstrated by a marked increase in minimum surface tension (Figure 8D), which translates *in vivo* to reduced surface tension lowering activity and reduced overall surfactant function. The airspace of the injured lung contained eightfold more protein (Figure 8C), which likely represents a combination of proteins released by the lung parenchyma and serum protein extravasation across a disrupted alveolocapillary barrier.

## Discussion

The macroscale pressures and flows applied at the trachea during mechanical ventilation result in microscale parenchymal injury (Dreyfuss and Saumon, 1998). The resulting ingress of protein-rich edema into the parenchymal airspace causes changes in alveolar dynamics at the microscale that are then reflected in macroscale alterations in lung function (Hamlington et al., 2018b). Macro- to micro-scale interactions in VILI thus take place in both directions, which we investigated by correlating VILI-induced changes in mouse lung microstructure to changes in lung function. Understanding the link between structure and function at both micro and macro levels of scale is crucial for developing optimized mechanical ventilation strategies that interrupt the vicious cycle of surfactant degradation and inactivation (Agassandian and Mallampalli, 2013), mechanical injury, inflammation, and altered lung function that drives VILI pathogenesis.

The most striking alterations in VILI-related organ-scale function are seen at low lung volumes (Figure 1, column 1), where we observe an approximately twofold increase in the first value of elastance measured at PEEP = 0 cmH_2_O immediately following an RM in VILI compared to controls (Table 1, intercept). This is attributable to the loss of roughly half the ventilated alveoli [N(alv), Figure 6B], due both to derecruitment that is reversible with an RM and the flooding of alveoli [N(flood), Figure 6C] that are not recruitable. H then continues to increase over the subsequent three minutes of $D_{rec}^{PEEP0}$ at a rate that is greater in the injured lungs (Table 1, slope). Although we cannot assess time-dependent alveolar collapse in fixed tissue, our previous studies indicate that this increased rate of lung stiffening is a consequence of accelerated alveolar derecruitment (Smith et al., 2015) that we would ascribe, in the current study, to elevated minimum surface tension (Figure 8D).

The PEEP = 3 and 6 cmH_2_O derecruitability tests also show progressive increases in H, although of substantially smaller magnitudes than at PEEP = 0 cmH_2_O (Table 1), no doubt because airway pressures remained above the derecruitment pressures of most of the unstable alveoli at the higher PEEP levels. This is borne out by our stereological analysis, which shows a decrease in N(alv) at the higher PEEP levels that is commensurate with the corresponding rates of increase in H. However, the total number of alveoli, N(alv,total), which includes both patent and flooded units, was unchanged in the VILI animals compared to controls at Paw = 5 and 10 cmH_2_O. This indicates that the increase in elastance in $D_{rec}^{PEEP3}$ and $D_{rec}^{PEEP6}$ is primarily due to the accumulation of flooded, non-recruitable alveoli rather than recruitment-resistant atelectasis or small airway closure. Figure 7 shows that the H values measured at the end of the derecruitability tests in all animals are inversely related to the fraction of patent alveoli (the “open fraction”), as we have previously postulated in our modeling studies of ventilator and acute lung injury in rodents (Smith et al., 2013, 2015; Hamlington et al., 2016b). This type of relationship was also observed during the acute phase of bleomycin injury in rats (Lutz et al., 2015; Knudsen et al., 2018) and lends strong support to the notion that the increases in lung stiffness seen in the acutely injured lung are largely reflective of loss of lung units through various derecruitment mechanisms. This has the important corollary that the dynamics of recruitment and derecruitment in the injured lung may be inferred directly from observed changes in H.

As expected, the loss of ventilated alveoli with VILI was accompanied by corresponding losses in V(alvair) at all pressures (Figure 5A). Less expected was the reduction in *V*_n_(alv) (Figure 6E), because we previously observed mean alveolar volume to increase during the first 3 days of bleomycin injury in rats (Knudsen et al., 2018). Bleomycin-treated rats experience similar changes in minimum surface tension (Lutz et al., 2015) to those we found in the present study, so the differences in alveolar volume behavior cannot be attributed to differences in surfactant function. On the other hand, there may have been differences in the way that alveoli became derecruited in the two models. Finite element simulations indicate that the degree of alveolar enlargement caused by tethering forces from adjacent derecruited regions of the lung is greater when the adjacent alveoli are collapsed as opposed to being merely flooded (Albert et al., 2019). The later stages of bleomycin injury are characterized by widespread alveolar collapse that is resistant to reopening even at high airway pressures (Knudsen et al., 2018), which may explain why the mean volume of open alveoli was increased in this model. In contrast, in the VILI model of the present study the collapsed alveoli could be reopened by an RM, and the alveoli remained patent down to Paw = 5 cmH_2_O, which would have reduced the tethering forces. Furthermore, at Paw = 2 cmH_2_O the VILI lungs were characterized by extensive areas of both flooded and collapsed alveoli interspersed with regions of normal-appearing alveoli, so that tethering-induced alveolar volume increases would be confined to the boundaries between the collapsed and open regions.

The surface area available for gas exchange (Figure 5D) followed the same trend as V(alvair), increasing with Paw and decreasing with VILI. However, the mean surface area-to-volume ratio of an individual alveolus (Figure 6F) showed a striking increase with VILI at Paw = 2 cmH_2_O, indicating that the alveoli themselves had become less spherical and the alveolar septa possibly more convoluted. This is generally what one might expect in a deflating alveolus (Knudsen and Ochs, 2018), particularly if the alveolar walls become stiffer in VILI. Alveolar wall thickness was essentially unchanged by VILI (Figure 6A), however, so the stiffening was more likely due to increased surface tension.

The effect of alveolar stiffness on macro-scale mechanics was secondary to the loss of ventilated alveoli due to collapse and flooding (Figure 7). Our data indicate that the degradation of mechanical function with VILI follows the same path as in ARDS where different alveolar phenotypes have been postulated to define three distinct lung zones (Gattinoni et al., 1987): Zone D is comprised of damaged alveoli that cannot be recruited, corresponding to N(flood) in the current study. Zone R is comprised of unstable alveoli that are injured but recruitable, represented by the decrease in N(alv) between Paw = 5 and 2 cmH_2_O in VILI (Figure 6D). This represents an VILI-induced upward shift of the distribution of alveolar derecruitment pressures (i.e., alveolar instability) since there is no change in N(alv) between Paw = 5 and 2 cmH_2_O in the controls. Zone H comprises the remaining open alveoli (i.e., the “baby lung”) that remain aerated at low Paw. Our microscale analysis shows that the mechanics of these open alveoli are not entirely normal since *V*_n_(alv) and S/V(alv) are altered in VILI, but these alterations are secondary contributors to organ-scale mechanics when compared to the loss of ventilated alveoli. These three alveolar phenotypes have different dynamic behaviors that are reflected in the time- and pressure-dependence of H. It is important to note that while these three alveolar phenotypes are generally stable there may be variability between respiratory cycles. *In vivo* confocal microscopy shows that flooded alveoli are typically stable but may occasionally expel their edematous contents and become re-aerated (Wu et al., 2017). Likewise, nearby alveoli may alternately collapse on subsequent respiratory cycles (Broche et al., 2017) so that on subsequent breaths a single alveolus my either be collapsed or open.

The correlation between alveolar recruitment and elastance (Figure 7) may provide a mechanistic basis for identifying optimally lung-protective ventilation strategies. Figure 6 shows alveolar recruitment occurring between pressures of 2 and 5 cmH_2_O and, as such, H decreases as PEEP is increased from 0 to 3 cmH_2_O (Figure 1). By contrast, further increasing PEEP to 6 cmH_2_O causes a modest increase in elastance (and thus driving pressure) that is indicative of parenchymal overdistension. Taken together, these data suggest that the optimal PEEP based on lung elastance (or driving pressure) for these VILI mice lies between 3 and 6 cmH_2_O to recruit the recruitable alveoli (Zone R) without causing tissue overdistension by applying excessive PEEP in a futile attempt to recruit the Zone D alveoli that, in our VILI model, are flooded with edema.

Tissue damping is challenging to interpret through the lens of fixed tissue because it describes the viscous losses in the parenchyma that are, by definition, dynamic processes. The fractional changes in G during the derecruitability tests were, for the most part, rather similar to the corresponding fractional changes in H (Table 1), which is most readily interpreted as being due simply to derecruitment of lung units, although it could also reflect an intrinsic coupling of the elastic and dissipative properties of lung parenchyma (Kaczka and Smallwood, 2012). Nevertheless, the largest alteration in G caused by VILI was an approximately twofold increase during $D_{rec}^{PEEP0}$ (Figure 2D) that was slightly larger than the corresponding relative increase in H (Figure 1D). This may have been due to an increase in some form of dissipative process in the lung, such as intra-tidal recruitment (Kaczka et al., 2005, 2011), movement of fluid within and between partially flooded alveoli, or the folding and unfolding of septal pleats as suggested by an increased S/V(alv). In contrast, at PEEP = 3 and 6 cmH_2_O the VILI-induced changes in G were relatively less than those in H. This contrasts with findings in open-chest but otherwise healthy mice in which tissue hysteresivity = G/H remained constant with lung volume (Sly et al., 2003). Theoretical analysis, however, shows that reductions in hysteresivity can arise if ongoing recruitment and derecruitment of lung units occurs while lung impedance is being measured by the FOT (Bates and Allen, 2006), as might easily be the case in severely injured lungs.

The decrease in airway resistance with PEEP (Figure 3) is readily attributable to the increase in airway caliber caused by airway-parenchymal tethering forces. This mechanism may also explain the modest VILI-induced decrease in Rn during $D_{rec}^{PEEP6}$, since flooded alveoli near the airways would be unable to expand normally during lung inflation and thus would cause tethering-forces on the airway to be correspondingly increased. Similarly, reduced or compressive tethering forces in atelectatic regions might explain why Rn was increased after VILI during $D_{rec}^{PEEP0}$. However, these changes in Rn were relatively minor, and were even non-existent during $D_{rec}^{PEEP3}$, indicating that central airway structure was not substantially affected by injurious ventilation. Indeed, stereological analysis revealed only occasional edema in the airways, and we did not find any airways that were either partially buckled or fully collapsed.

The structural and functional changes detailed above are associated with changes to the alveolar fluid-mechanical microenvironment that oppose alveolar inflation and promote collapse. The increased volume of fluid (Figure 5F) in partially flooded alveoli decreases the interfacial radius of curvature, which increases the pressure differential across the air–liquid interface and causes the lining fluid to exert a greater inward force that facilitates collapse. Furthermore, the functionality of the surfactant system is compromised (Figure 8C) from an apparent increase in surfactant degradation (increased SA fraction and decreased LA/SA ratio) and inactivation of PLs in the LA aggregates. The degradation of surface-active LA (Yamada et al., 1990) into surface-inactive SA (Figure 8A) may be attributed to large cyclic strains imposed by high-volume, PEEP = 0 cmH_2_O ventilation (Greenfield et al., 1964) that severely compresses the interfacial film so that it ruptures on re-expansion (Wyszogrodski et al., 1975). These mechanical effects can occur in the first 5 min of high tidal-volume ventilation (McClenahan and Urtnowski, 1967; Forrest, 1972). Therefore, in the present study, ventilation-induced surfactant degradation may have been the first step in the vicious cycle of altered alveolar dynamics, collapse, atelectrauma, and volutrauma that lead to eventually fatal VILI.

The mechanism for inactivation of the LA fraction is less clear and may very well be multifactorial. Airspace protein content is elevated (Figure 8C) due to alveolar surface damage (Dreyfuss et al., 1988; Hamlington et al., 2018b) and the resulting alveolocapillary leak (Hamlington et al., 2018a). Alveolar surface damage increases the BALF concentration of epithelial proteins such as E-cadherin while alveolocapillary leak accounts for the presence of serum proteins including albumin and immunoglobulin (IgG) in the BALF of mice ventilated under similar conditions (Smith et al., 2017). Our surface tension analysis includes the effects of BAL proteins that migrate with the PLs of the LA fraction during ultracentrifugation and inhibit surface lowering activity through disruption of phospholipid packing in the molecular films at the air–liquid interface. Furthermore, changes to the LA composition including phospholipids, surfactant-associate proteins or both may be present, and were not explored in these studies. Preliminary studies examining the presence of secretory phospholipases capable of targeting surfactant PLs revealed no significant changes in the BAL during this VILI model (data not shown) (Hite et al., 1998).

Our study has a number of limitations. First, the lungs were prepared for morphometric analysis with the chest wall retracted, which means that at high inflation pressures the lung volumes would have been greater than in closed-chest animals due to absence of constraint by the thorax. Conversely, at low inflation pressures the absence of the chest wall would have resulted in lower lung volumes due to the absence of the negative pleural pressure that opposes collapse. Based on lung volumes, airway pressures, and esophageal pressures measured in mice of equivalent age and weight to those used in the current study (Lai and Chou, 2000) we estimate that our morphometry data recorded at Paw = 2 cmH_2_O corresponds to Paw = 1.5 cmH_2_O in the intact mouse, an open-chested Paw = 10 cmH_2_O is equivalent to 12 cmH_2_O with the chest wall present, and at Paw = 5 cmH_2_O the contribution of the chest wall is approximately zero. In addition, our morphometry was conducted on fixed tissue sections and this limits the analysis to static conditions following a prolonged breath hold. As such, we are unable to offer any insights into the intra-breath alveolar and acinar dynamics, which could provide important insights into, for example, how the elastance of an individual alveolus is altered by VILI. Phase contrast synchrotron computed tomography offers a pathway to analyze alveolar dynamics in future studies (Chang et al., 2015; Morgan et al., 2020). Another limitation is that the high inspiratory pressures and zero PEEP used to generate VILI in the current study are far outside clinical guidelines for safe ventilation. Indeed, they were specifically chosen to provide the volutrauma and atelectrauma necessary for VILI pathogenesis, while reducing the potential for deleterious metabolic effects occurring over long periods of ventilation (Wilson and Takata, 2019). Accordingly, our analysis is limited to the direct effects of VILI without the contribution of downstream inflammatory effects that take longer to manifest. Finally, the fact that our analysis was conducted in mice must be considered when applying our findings to ARDS and VILI in human subjects. Since the mouse lung is quite small, the effect of the gravitational gradient on lung structure and function is reduced in comparison to human subjects. Intrinsic structural differences are also present, including the pattern of branching which is monopodial (asymmetric) in mice and dichotomous (symmetric) in humans.

## Conclusion

Three alveolar phenotypes typically develop with VILI: (1) flooded alveoli that cannot be recruited at any pressure, (2) unstable alveoli that are open at high pressures but readily collapse as pressure is reduced, and (3) relatively normal alveoli that remain open at low pressures. The relative proportions of each phenotype can be estimated using derecruitability tests that quantify time-dependent alveolar derecruitment as a function of PEEP. These correlations between lung structure and function could provide insights into optimally lung-protective ventilation strategies.

## Data Availability Statement

The datasets generated for this study are available on request to the corresponding author.

## Ethics Statement

The animal study was reviewed and approved by University of Vermont, Institutional Animal Care and Use Committee and the University of Colorado Denver Anschutz Medical Campus, Institutional Animal Care and Use Committee.

## Author Contributions

BS, GR, KH, LK, MO, RH, and JB contributed to the conception and design of the study. BS, GR, AC, CM, KO, CC, and MN acquired the experimental data. BS, KH, RH, and JB analyzed and interpreted the data. BS drafted the manuscript. CM, KO, RH, and JB wrote sections of the manuscript. All authors contributed to the article and approved the submitted version.

## Conflict of Interest

The authors declare that the research was conducted in the absence of any commercial or financial relationships that could be construed as a potential conflict of interest.

## References

[B1] Agassandian M. Mallampalli R. K. (2013). Surfactant phospholipid metabolism. *Biochim. Biophys. Acta* 1831 612–625. 10.1016/j.bbalip.2012.09.010 23026158 PMC3562414

[B2] Albert R. K. Smith B. Perlman C. E. Schwartz D. A. (2019). Is progression of pulmonary fibrosis due to ventilation-induced lung injury?. *Am. J. Respir. Crit. Care Med*. 200 140–151. 10.1164/rccm.201903-0497PP 31022350 PMC6635778

[B3] Aoyama H. Pettenuzzo T. Aoyama K. Pinto R. Englesakis M. Fan E. (2018). Association of driving pressure with mortality among ventilated patients with acute respiratory distress syndrome: a systematic review and meta-analysis. *Crit. Care Med.* 46 300–306. 10.1097/ccm.0000000000002838 29135500

[B4] Bachofen H. Ammann A. Wangensteen D. Weibel E. R. (1982). Perfusion fixation of lungs for structure-function analysis: credits and limitations. *J. Appl. Physiol. Respir. Environ. Exerc. Physiol.* 53 528–533. 10.1152/jappl.1982.53.2.528 6181045

[B5] Bates J. H. Allen G. B. (2006). The estimation of lung mechanics parameters in the presence of pathology: a theoretical analysis. *Ann. Biomed. Eng.* 34 384–392. 10.1007/s10439-005-9056-6 16468093

[B6] Broche L. Perchiazzi G. Porra L. Tannoia A. Pellegrini M. Derosa S. (2017). Dynamic mechanical interactions between neighboring airspaces determine cyclic opening and closure in injured lung. *Crit. Care Med.* 45 687–694. 10.1097/Ccm.0000000000002234 28107207 PMC5702254

[B7] Chang S. Kwon N. Kim J. Kohmura Y. Ishikawa T. Rhee C. K. (2015). Synchrotron X-ray imaging of pulmonary alveoli in respiration in live intact mice. *Sci. Rep.* 5:8760. 10.1038/srep08760 25737245 PMC4348649

[B8] Dreyfuss D. Saumon G. (1998). Ventilator-induced lung injury: lessons from experimental studies. *Am. J. Respir. Crit. Care Med.* 157 294–323. 10.1164/ajrccm.157.1.9604014 9445314

[B9] Dreyfuss D. Soler P. Basset G. Saumon G. (1988). High inflation pressure pulmonary edema. Respective effects of high airway pressure, high tidal volume, and positive end-expiratory pressure. *Am. Rev. Respir. Dis.* 137 1159–1164. 10.1164/ajrccm/137.5.1159 3057957

[B10] Enhorning G. Holm B. A. (1993). Disruption of pulmonary surfactant’s ability to maintain openness of a narrow tube. *J. Appl. Physiol.* 74 2922–2927. 10.1152/jappl.1993.74.6.2922 8365993

[B11] Force A. D. T. Ranieri V. M. Rubenfeld G. D. Thompson B. T. Ferguson N. D. Caldwell E. (2012). Acute respiratory distress syndrome: the Berlin Definition. *JAMA* 307 2526–2533. 10.1001/jama.2012.5669 22797452

[B12] Forrest J. B. (1972). The effect of hyperventilation on pulmonary surface activity. *Br. J. Anaesth.* 44 313–320. 10.1093/bja/44.4.313 4555708

[B13] Gattinoni L. Pesenti A. Avalli L. Rossi F. Bombino M. (1987). Pressure-volume curve of total respiratory system in acute respiratory failure. Computed tomographic scan study. *Am. Rev. Respir. Dis.* 136 730–736. 10.1164/ajrccm/136.3.730 3307572

[B14] Gil J. Bachofen H. Gehr P. Weibel E. R. (1979). Alveolar volume-surface area relation in air- and saline-filled lungs fixed by vascular perfusion. *J. Appl. Physiol. Respir. Environ. Exerc. Physiol.* 47 990–1001. 10.1152/jappl.1979.47.5.990 511725

[B15] Greenfield L. J. Ebert P. A. Benson D. W. (1964). Effect of positive pressure ventilation on surface tension properties of lung extracts. *Anesthesiology* 25 312–316. 10.1097/00000542-196405000-00009 14156571

[B16] Hamlington K. L. Bates J. H. T. Roy G. S. Julianelle A. J. Charlebois C. Suki B. (2018a). Alveolar leak develops by a rich-get-richer process in ventilator-induced lung injury. *PLoS One* 13:e0193934. 10.1371/journal.pone.0193934 29590136 PMC5874026

[B17] Hamlington K. L. Smith B. J. Dunn C. M. Charlebois C. M. Roy G. S. Bates J. H. T. (2018b). Linking lung function to structural damage of alveolar epithelium in ventilator-induced lung injury. *Respir. Physiol. Neurobiol.* 255 22–29. 10.1016/j.resp.2018.05.004 29742448 PMC5986619

[B18] Hamlington K. L. Dunn C. M. Roy G. S. Smith B. J. Bates J. H. T. (2016a). Linking alveolar epithelial barrier disruption to function in ventilator-induced lung injury. *Am. J. Respir. Crit. Care Med.* 193:A4826.

[B19] Hamlington K. L. Smith B. J. Allen G. B. Bates J. H. (2016b). Predicting ventilator-induced lung injury using a lung injury cost function. *J. Appl. Physiol.* 121 106–114. 10.1152/japplphysiol.00096.2016 27174922 PMC4967254

[B20] Hantos Z. Daroczy B. Suki B. Nagy S. Fredberg J. J. (1992). Input impedance and peripheral inhomogeneity of dog lungs. *J. Appl. Physiol.* 72 168–178. 10.1152/jappl.1992.72.1.168 1537711

[B21] Hernandez L. A. Peevy K. J. Moise A. A. Parker J. C. (1989). Chest wall restriction limits high airway pressure-induced lung injury in young-rabbits. *J. Appl. Physiol.* 66 2364–2368. 10.1152/jappl.1989.66.5.2364 2745302

[B22] Hite R. D. Seeds M. C. Bowton D. L. Grier B. L. Safta A. M. Balkrishnan R. (2005). Surfactant phospholipid changes after antigen challenge: a role for phosphatidylglycerol in dysfunction. *Am. J. Physiol. Lung Cell Mol. Physiol.* 288 L610–L617. 10.1152/ajplung.00273.2004 15347567

[B23] Hite R. D. Seeds M. C. Jacinto R. B. Balasubramanian R. Waite M. Bass D. (1998). Hydrolysis of surfactant-associated phosphatidylcholine by mammalian secretory phospholipases A2. *Am. J. Physiol.* 275 L740–L747. 10.1152/ajplung.1998.275.4.L740 9755106

[B24] Hsia C. C. Hyde D. M. Ochs M. Weibel E. R. (2010). Ats Ers Joint Task Force on Quantitative Assessment of Lung Structure. An official research policy statement of the American Thoracic Society/European Respiratory Society: standards for quantitative assessment of lung structure. *Am. J. Respir. Crit. Care Med.* 181 394–418. 10.1164/rccm.200809-1522ST 20130146 PMC5455840

[B25] Kaczka D. W. Cao K. L. Christensen G. E. Bates J. H. T. Simon B. A. (2011). Analysis of regional mechanics in canine lung injury using forced oscillations and 3d image registration. *Ann. Biomed. Eng.* 39 1112–1124. 10.1007/s10439-010-0214-0 21132371 PMC3036832

[B26] Kaczka D. W. Hager D. N. Hawley M. L. Simon B. A. (2005). Quantifying mechanical heterogeneity in canine acute lung injury: impact of mean airway pressure. *Anesthesiology* 103 306–317. 16052113 10.1097/00000542-200508000-00014

[B27] Kaczka D. W. Smallwood J. L. (2012). Constant-phase descriptions of canine lung, chest wall, and total respiratory system viscoelasticity: effects of distending pressure. *Respir. Physiol. Neurobiol.* 183 75–84. 10.1016/j.resp.2012.06.008 22691447 PMC3409308

[B28] Knudsen L. Lopez-Rodriguez E. Berndt L. Steffen L. Ruppert C. Bates J. H. T. (2018). Alveolar Micromechanics in Bleomycin-induced Lung Injury. *Am. J. Respir. Cell Mol. Biol.* 59 757–769. 10.1165/rcmb.2018-0044OC 30095988 PMC6293074

[B29] Knudsen L. Ochs M. (2018). The micromechanics of lung alveoli: structure and function of surfactant and tissue components. *Histochem. Cell Biol.* 150 661–676. 10.1007/s00418-018-1747-9 30390118 PMC6267411

[B30] Lai Y. L. Chou H. (2000). Respiratory mechanics and maximal expiratory flow in the anesthetized mouse. *J. Appl. Physiol.* 88 939–943. 10.1152/jappl.2000.88.3.939 10710389

[B31] Lenth R. V. (2016). Least-Squares Means: the R Package lsmeans. *J. Stat. Softw.* 69 1–33. 10.18637/jss.v069.i01

[B32] Lutz D. Gazdhar A. Lopez-Rodriguez E. Ruppert C. Mahavadi P. Günther A. (2015). Alveolar derecruitment and collapse induration as crucial mechanisms in lung injury and fibrosis. *Am. J. Respir. Cell Mol. Biol.* 52 232–243. 10.1165/rcmb.2014-0078oc 25033427

[B33] McClenahan J. B. Urtnowski A. (1967). Effect of ventilation on surfactant, and its turnover rate. *J. Appl. Physiol.* 23 215–220. 10.1152/jappl.1967.23.2.215 6031191

[B34] Mellenthin M. M. Seong S. A. Roy G. S. Bartolak-Suki E. Hamlington K. L. Bates J. H. T. (2019). Using injury cost functions from a predictive single-compartment model to assess the severity of mechanical ventilator-induced lung injuries. *J. Appl. Physiol.* 127 58–70. 10.1152/japplphysiol.00770.2018 31046518 PMC6692741

[B35] Morgan K. S. Parsons D. Cmielewski P. McCarron A. Gradl R. Farrow N. (2020). Methods for dynamic synchrotron X-ray respiratory imaging in live animals. *J. Synchrotr. Radiat.* 27(Pt 1), 164–175. 10.1107/S1600577519014863 31868749 PMC6927518

[B36] Morton S. E. Knopp J. L. Chase J. G. Moller K. Docherty P. Shaw G. M. (2019). Predictive virtual patient modelling of mechanical ventilation: impact of recruitment function. *Ann. Biomed. Eng.* 47 1626–1641. 10.1007/s10439-019-02253-w 30927170

[B37] Mühlfeld C. Knudsen L. Ochs M. (2013). “Stereology and morphometry of lung tissue,” in *Methods in Molecular Biology*, eds Taatjes D. J. Mossman B. T., (Totowa, NJ: Humana Press). 10.1007/978-1-62703-056-4_18 23027012

[B38] Muscedere J. G. Mullen J. B. M. Gan K. Slutsky A. S. (1994). Tidal Ventilation at Low Airway Pressures Can Augment Lung Injury. *Am. J. Respir. Crit. Care Med.* 149 1327–1334. 10.1164/ajrccm.149.5.8173774 8173774

[B39] Ochs M. Nyengaard L. Jung A. Knudsen L. Voigt M. Wahlers T. (2004). The number of alveoli in the human lung. *Am. J. Respir. Crit. Care Med.* 169 120–124. 10.1164/rccm.200308-1107OC 14512270

[B40] Pereira Romano M. L. Maia I. S. Laranjeira L. N. Damiani L. P. Paisani D. M. Borges M. C. (2020). Driving pressure-limited strategy for patients with acute respiratory distress syndrome. A pilot randomized clinical trial. *Ann. Am. Thorac. Soc.* 17 596–604. 10.1513/AnnalsATS.201907-506OC 32069068

[B41] Pinheiro J. C. Bates D. M. (2000). *Mixed-Effects Models in S and S-PLUS.* New York, NY: Springer.

[B42] Rouser G. Fleischer S. Yamamoto A. (1970). Two dimensional thin layer chromatographic separation of polar lipids and determination of phospholipids by phosphorus analysis of spots. *Lipids* 5 494–496. 10.1007/bf02531316 5483450

[B43] Schneider J. P. Ochs M. (2014). Alterations of mouse lung tissue dimensions during processing for morphometry: a comparison of methods. *Am. J. Physiol. Lung Cell Mol. Physiol.* 306 L341–L350. 10.1152/ajplung.00329.2013 24375800

[B44] Sly P. D. Collins R. A. Thamrin C. Turner D. J. Hantos Z. (2003). Volume dependence of airway and tissue impedances in mice. *J. Appl. Physiol.* 94 1460–1466. 10.1152/japplphysiol.00596.2002 12391040

[B45] Smith B. J. Bartolak-Suki E. Suki B. Roy G. S. Hamlington K. L. Charlebois C. M. (2017). Linking ventilator injury-induced leak across the blood-gas barrier to derangements in murine lung function. *Front. Physiol.* 8:466. 10.3389/fphys.2017.00466 28736528 PMC5500660

[B46] Smith B. J. Grant K. A. Bates J. H. (2013). Linking the development of ventilator-induced lung injury to mechanical function in the lung. *Ann. Biomed. Eng.* 41 527–536. 10.1007/s10439-012-0693-2 23161164 PMC3600072

[B47] Smith B. J. Lundblad L. K. Kollisch-Singule M. Satalin J. Nieman G. Habashi N. (2015). Predicting the response of the injured lung to the mechanical breath profile. *J. Appl. Physiol.* 118 932–940. 10.1152/japplphysiol.00902.2014 25635004 PMC4385881

[B48] Webb H. H. Tierney D. F. (1974). Experimental pulmonary-edema due to intermittent positive pressure ventilation with high inflation pressures. Protection by positive end-expiratory pressure. *Am. Rev. Respir. Dis.* 110 556–565. 4611290 10.1164/arrd.1974.110.5.556

[B49] Wilson M. Takata M. (2019). Mechanical ventilation in mice: does longer equal better? *Am. J. Respir. Cell Mol. Biol.* 60 137–138. 10.1165/rcmb.2018-0308ED 30304642

[B50] Wu Y. Nguyen T. L. Perlman C. E. (2017). Accelerated deflation promotes homogeneous airspace liquid distribution in the edematous lung. *J. Appl. Physiol.* 122 739–751. 10.1152/japplphysiol.00526.2016 27979983 PMC5407205

[B51] Wyszogrodski I. Kyei-Aboagye K. Taeusch H. W. Jr. Avery M. E. (1975). Surfactant inactivation by hyperventilation: conservation by end-expiratory pressure. *J. Appl. Physiol.* 38 461–466. 10.1152/jappl.1975.38.3.461 1097385

[B52] Yamada T. Ikegami M. Jobe A. H. (1990). Effects of surfactant subfractions on preterm rabbit lung function. *Pediatr. Res.* 27 592–598. 10.1203/00006450-199006000-00011 2356104

[B53] Yen S. Preissner M. Bennett E. Dubsky S. Carnibella R. O’Toole R. (2019). The link between regional tidal stretch and lung injury during mechanical ventilation. *Am. J. Respir. Cell Mol. Biol.* 60 569–577. 10.1165/rcmb.2018-0143OC 30428271

